# Brain rhythms from delayed interaction of fluctuations

**DOI:** 10.1186/1471-2202-15-S1-P114

**Published:** 2014-07-21

**Authors:** Alexandre Payeur, Leonard Maler, André Longtin

**Affiliations:** 1 Department of Physics, University of Ottawa, Ottawa, Canada, K1N 6N5; 2 Department of Cell and Molecular Medecine, University of Ottawa, Canada, K1H 8M5

## 

**Figure 1:**
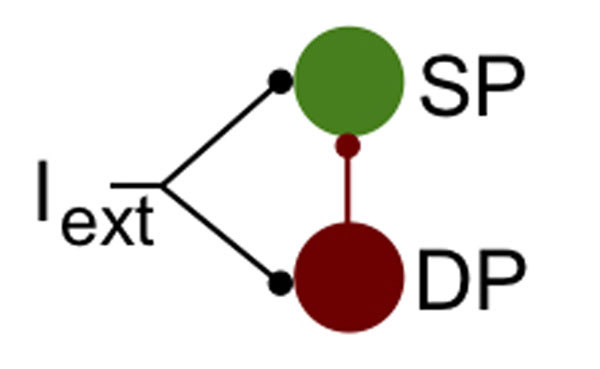
Schematics of the feedforward network. Populations labeled DP and SP both receive an input I_ext_. All DP neurons project onto all SP cells, but there are no recurrent connections within the respective populations.

Typically, neural oscillations appear in networks presenting some degree of recurrent connections and feedback. Recurrent connections between neurons may promote synchronization. In particular, networks of interconnected inhibitory neurons synchronize and constitute a potent substrate for gamma (~ 40 Hz) oscillations [1]. Also, delayed feedback augments the dimensionality of dynamical systems, allowing for oscillatory behaviors [2,3]. Here, we report neural oscillations in a feedforward circuit without recurrent connections. The network’s architecture was inspired by the physiology of the electrosensory lateral line lobe (ELL) of weakly electric fish, and has already been shown to possess efficient gain control mechanisms [4]. A population of inhibitory neurons is connected to another with a delay, with both populations receiving noisy inputs (Figure 1). When the same input was shared by all neurons the resulting stimulus-induced correlations led to rhythms in the inhibited population (SP in Figure 1). We used simulations of spiking neurons and linear response theory to show that the delayed interaction between the stimulus and the inhibition from the feedforward population was responsible for eliciting the oscillatory activity. These oscillations were robust to the presence of heterogeneities in both the neurons' properties and the axonal delays. Moreover, we implemented simple ON- and OFF-like receptive fields into both populations and showed that different connectivity patterns and synaptic polarities between ON and OFF neurons can either enhance or hinder oscillations in feedforward nets. For instance, we found that an ON (OFF) inhibitory population projecting onto an ON (OFF) population generated rhythms, but not OFF inhibitory neurons connected to ON neurons (or ON to OFF). This allowed us to draw conclusions on the possible connectivity schemes leading to rhythms in the ELL.
